# No superior treatment for primary osteochondral defects of the talus

**DOI:** 10.1007/s00167-017-4616-5

**Published:** 2017-06-27

**Authors:** Jari Dahmen, Kaj T. A. Lambers, Mikel L. Reilingh, Christiaan J. A. van Bergen, Sjoerd. A. S. Stufkens, Gino M. M. J. Kerkhoffs

**Affiliations:** 1 Department of Orthopedic Surgery, Academic Medical Centre, University of Amsterdam, Meibergdreef 9, 1105 AZ Amsterdam, The Netherlands; 2 Academic Center for Evidence based Sports medicine (ACES), Meibergdreef 9, 1105 AZ Amsterdam, The Netherlands; 3 Amsterdam Collaboration for Health and Safety in Sports (ACHSS), AMC/VUmc IOC Research Center, Meibergdreef 9, 1105 AZ Amsterdam, The Netherlands; 4 Department of Orthopedic Surgery, Amphia Hospital, Breda, The Netherlands

**Keywords:** Ankle, Talus, Osteochondral lesion, Defect, Systematic review, Arthroscopy, Articular cartilage, Success rate

## Abstract

**Purpose:**

The purpose of this systematic literature review is to detect the most effective treatment option for primary talar osteochondral defects in adults.

**Methods:**

A literature search was performed to identify studies published from January 1996 to February 2017 using PubMed (MEDLINE), EMBASE, CDSR, DARE, and CENTRAL. Two authors separately and independently screened the search results and conducted the quality assessment using the Newcastle–Ottawa Scale. Subsequently, success rates per separate study were calculated. Studies methodologically eligible for a simplified pooling method were combined.

**Results:**

Fifty-two studies with 1236 primary talar osteochondral defects were included of which forty-one studies were retrospective and eleven prospective. Two randomised controlled trials (RCTs) were identified. Heterogeneity concerning methodological nature was observed, and there was variety in reported success rates. A simplified pooling method performed for eleven retrospective case series including 317 ankles in the bone marrow stimulation group yielded a success rate of 82% [CI 78–86%]. For seven retrospective case series investigating an osteochondral autograft transfer system or an osteoperiosteal cylinder graft insertion with in total 78 included ankles the pooled success rate was calculated to be 77% [CI 66–85%].

**Conclusions:**

For primary talar osteochondral defects, none of the treatment options showed any superiority over others.

**Level of evidence:**

IV.

## Introduction

A talar osteochondral defect (OCD) is a combined lesion of the subchondral bone and its overlying cartilage and often has a severe impact on the quality of life of active patients [134]. The general consensus is that bone marrow stimulation (BMS) is administered for primary smaller defects. Other surgical options are internal fixation, osteochondral autograft transfer systems (OATS), chondrocyte implantation, retrograde drilling, metal resurfacing, total ankle prostheses or arthrodesis [44, 56, 124].

The effectiveness of the interventions varies greatly in the literature, and although a number of previous systematic reviews have been conducted, a definite treatment option regarded as *the* golden standard has yet to be identified [32, 69, 85, 119, 128, 135]. Additionally, prior systematic reviews either investigated sole treatment options or did not distinguish between primary and non-primary talar defects [32, 69, 85, 135]. Therefore, this could introduce a mispresentation of the reported success rates. Furthermore, the most comprehensive review by Zengerink et al. [135] included articles published up to 2006. Since then, a high number of articles investigating novel interventions for talar OCDs have been published [66, 94, 95, 122]. The aim of the present review is therefore to examine and compare the clinical effectiveness of all treatment strategies for exclusively primary talar OCDs in adults. The hypothesis is that no significant differences considering clinical outcome of these different treatment strategies are to be found. This study presents novel findings and gives novel insight into the clinical effectiveness of treatment strategies for primary talar osteochondral defects exclusively.

## Materials and methods

The systematic review was prospectively registered at the PROSPERO register [23].

### Search strategy

Electronic databases PubMed (MEDLINE), EMBASE, CDSR, DARE and CENTRAL were screened from January 1996 to February 2017 for potential suitable articles (Appendix 1). This time frame was chosen as by 1996 the arthroscopic techniques for treating talar OCDs were fully developed and established in the orthopaedic field [126]. The full search strategy for all electronic databases is outlined in Appendix 1. Backward citation chaining strategy was applied as an additional search technique.

### Eligibility criteria and study selection (Fig. 1)

**Fig. 1:**
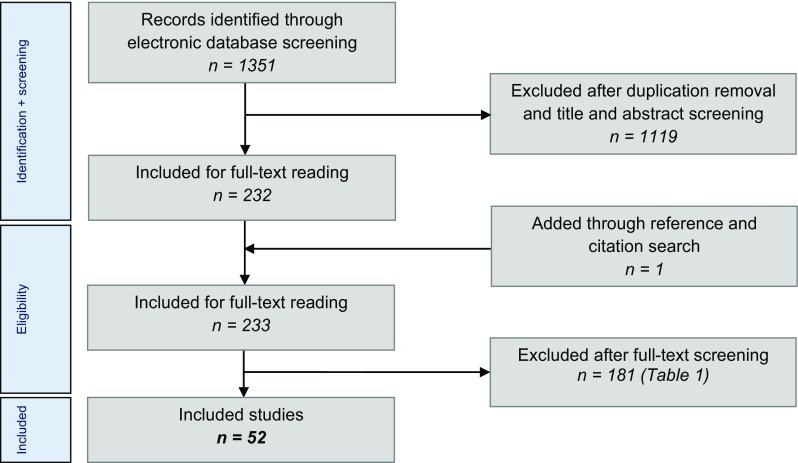
Literature selection algorithm—preferred reporting items for systematic reviews and meta-analyses (PRISMA)

Suitable randomised controlled trials (RCT) and observational studies assessing the effectiveness of all treatment strategies for primary talar OCDs in the adult patient population were included in the present study. The rationale for including non-randomised clinical studies is based on the substantial presence of the low-quality evidence research into talar osteochondral defects that has been conducted over the past two decades. The exclusion criteria for our review are presented in Table 1. When necessary, authors were contacted to provide separate data for patients with primary lesions only and/or for patients ≥18 years old. When no reply was reported, contact was sought by two reminder e-mails. If no response was recorded, the specific article was excluded. Independent evaluation of the articles and a subsequent discussion were performed by two reviewers (J.D. and K.L.) after title, abstract screening and full-text reading. In case of any disagreement after discussion, the opinion from an independent third investigator (G.K.) was decisive. Studies were not blinded for author, affiliation or source, and no limitations were put on language and publication status. The literature selection algorithm according to the preferred reporting items for systematic reviews and meta-analyses (PRISMA) is presented in Fig. 1 [67].

**Table 1:** Exclusion criteria

Exclusion criteria	No. of studies
Non-primary OCDs	91
<5 patients	20
Age: <18 years old	17
Patient overlap	14
Treatment inappropriately described	13
Combination of diagnoses (bipolar, fracture, etc.)	13
Combination of treatment groups and/or no separate data per group	8
Follow-up <6 months	2
Interpretable data not available	2
Asymptomatic lesion	1
Total no. of excluded studies	181

Some publications were excluded due to a combination of reasons

### Critical appraisal

A for-talar-OCD-modified Newcastle-Ottawa Scale (NOS) was utilised to assess the methodological quality (Appendix 3). Each included study was graded on methodological quality by two independent reviewers (J.D. and K.L.). When there was no agreement on the number of stars graded, assessment by an independent third investigator (G.K.) was decisive.

### Data extraction

By means of a standardised extraction form, data from the articles were extracted on study characteristics. Data on patient characteristics were retrieved and included age, gender, number of patients and ankles, symptom duration, location, side, size and stage of the defect according to a specifically reported OCD classification system, clinical scoring system utilised, history of ankle trauma and follow-up duration. Pre-operative and post-operative clinical outcome scores were extracted on mean scores, subjective satisfaction and number of patients treated successfully. The treatment strategy in question was defined to be successful when a good or excellent result at follow-up was reported, in combination with an accepted scoring system. The results were incorporated into the scoring system of Thompson and Loomer [118] (Appendix 2) when separate patient data were available though no success rates of specific treatment strategies were included. An ankle was considered to be successfully treated when at latest follow-up a post-operative AOFAS score at or above 80 was reached [59]. In case of the FAAM (Foot and Ankle Ability Measure) score, a percentage of 80 or higher was regarded to be a successful treatment [75].

### Statistical and data analysis

In case of identifying studies with highly differing methodological natures, a formal meta-analysis will not be performed. It will be decided upon visualising the results per study by means of a forest plot. If possible, a simplified pooling method will be used to combine data from different studies describing the results of similar treatment groups research by means of analogous methodologies. 95% binomial proportion confidence intervals for the success percentages of each study and the pooled studies will be calculated with the Wilson score interval and included in the forest plots (*CIA, Confidence Interval Analysis for Windows, version 2.2.0*) [19].

## Results

### Search results

**Fig. 2:**
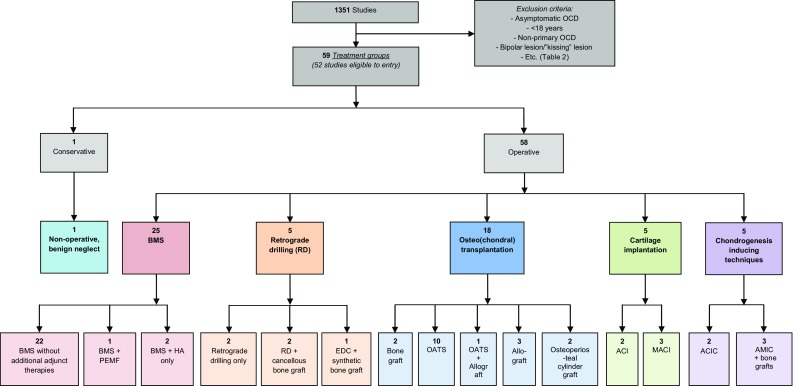
Flow chart of study inclusion and treatment of talar OCDs between 1996 and 2017. *ACI* autologous chondrocyte implantation, *ACIC* autologous collagen-induced chondrogenesis, *AMIC* autologous matrix-induced chondrogenesis, *RD* retrograde drilling, *BMS* bone marrow stimulation, *MACI* matrix-associated chondrocyte implantation, *OATS* osteochondral autograft transfer system, *HA* hyaluronic acid, *PEMF* pulsed electromagnetic fields, *ECD* endoscopic core decompression

The literature search yielded 1351 articles, and after title and abstract screening, 232 potentially suitable articles were included for full-text reading (Fig. 2). One study was added through reference and citation search. In total, 127 authors were contacted to request data according to our inclusion criteria. Subsequently, 33 studies could be included and 31 had to be excluded attributable to the extensive author contact process. In total, 181 publications had to be excluded due to a variety and combination of reasons (Table 1). This left 52 studies in total.

After screening and discussion between the first two authors there was overall consensus in all cases except for four where disagreement persisted. These were resolved by discussion with the senior author (G.K.).

Full consensus was reached between the reviewers regarding grading of methodological quality.

### Evaluation of the characteristics of included studies

A total of 1236 primary talar OCDs were included in the 52 studies. The average age was 36 [range 18–77], and the percentage of females and males was 34 and 66%, respectively. The right ankle was involved in 54% of the cases and the left ankle in 46%. The percentages of medial, lateral, central and combined medial and lateral location involvement were 77, 21, 2 and 0.4%, respectively. In 71% of the patients, a history of ankle trauma was reported. The most frequently used clinical scoring system and osteochondral damage classification system were the AOFAS and the Berndt and Harty Classification system, respectively [15, 59]. In total 25 different types of clinical scoring systems (Table 2) [9, 10, 15, 21, 35, 39, 48, 51, 59, 62, 71, 72, 75, 76, 82, 83, 86, 90, 100, 105, 109, 116, 117, 131] and 18 different utilised osteochondral damage classification systems were found (Table 3) [6, 15, 18, 30, 34, 37, 42, 46, 52, 73, 79, 87, 97, 106, 107, 125]. Data were extracted on the combined Berndt and Harty [15] and Loomer [68] stages for 257 ankles: there were 56 (22%), 68 (27%), 70 (27%), 37 (14%) and 26 (10%) Berndt and Harty [15] stage I, II, III, IV and V cases, respectively. Lastly, the mean of the follow-up time ranged from 6 to 143 months.

**Table 2:** Clinical scoring systems utilised for treatment of talar OCDs and associated knee scores in case of implantation techniques

Clinical scoring system	No. of studies
AOFAS Ankle/Hindfoot Scale [59]	43
VAS (Visual Analog Scale) [21]	27
Patient Satisfaction Score	17
Tegner score [116]	3
Short Form-36 [131]	4
Hannover score [117]	3
Freiburg Ankle Score [62]	3
Criteria proposed by Berndt and Harty [15]	3
Ogilvie Harris Score [90]	3
Ankle Activity Score [48]	2
Modified Cincinnati Knee Rating System [35]	2
Hospital of Special Surgery Patella Score [9]	2
IKDC Subjective and Objective Knee Evaluation Form [51, 86]	2
Clinical evaluation proposed by Shearer and Loomer [109]	1
RTA (Return to Activity) [105]	1
NRS (Numeric Rating Scale for pain and satisfaction) [39, 100]	1
Saxena criteria [105]	1
FAAM (Foot and Ankle Ability Measure) [75]	1
McCullough Score [76]	1
Foot Functioning Index [83]	1
MODEMS AAOS Foot and Ankle Follow-up Questionnaire [82]	1
Modified Cincinnati Knee Documentation Rating [72]	1
Bandi Knee Global Assessment Score [10]	1
Lysholm [71]	1
Foot and Ankle Disability Index (FADI) [49]	1

Some studies utilised >1 scoring system

**Table 3:** Classification systems utilised for osteochondral damage staging assessment

Classification systems	No. of studies
Berndt and Harty Classification System [15]	16
MOCART [73]	8
International Cartilage Repair Society (ICRS) [18]	8
Hepple et al. [52]	5
Ferkel and Cheng [37]	3
Anderson et al. [6]	3
Dipaola et al. [30]	3
Outerbridge Classification System [87]	2
Bristol Classification System [101]	2
Osteoarthritis Classification System [125]	1
Sefton Articular Stability Scale [107]	1
Pritsch Classification System [97]	1
FOC (Fracture, Osteonecrosis, Cyst) [34]	1
Takakura Radiologic Arthrosis Classification System [113]	1
Giannini Classification System [42]	1
Scranton and McDermott Classification System [106]	1
Mintz et al. [79]	1
Guhl [46]	1

Some studies utilised >1 classification system, and others did not utilise a classification system

### Methodological quality

The fifty-two publications altogether scored 182 stars out of maximum 260 stars (Table 4). Forty-one studies were assessed to be retrospectively conducted, and all studies except for two were conducted according to the study protocol. Therefore, all studies together scored a total number of 65 stars (max. = 104) on study design. Regarding the selection procedure, 43 out of 52 stars were scored in total, indicating that most studies reported a representative talar OCD patient population. Seventy-four out of 104 stars were scored on the outcome part of the adjusted Newcastle–Ottawa Scale. Independent blind assessment was performed in none of the studies, and in all except for one study outcome was assessed through record linkage. Numerical star outcomes on adequacy of follow-up of series were not uniform across the included studies.

**Table 4:** Table presenting the separate results of the adjusted Newcastle–Ottawa Scale

Category in question	Number of stars	Maximum number of stars	Proportion
Study design	65	104	65/104 = 63%
Selection	43	52	43/52 = 83%
Outcome	74	104	74/104 = 71%
Total	182	260	182/260 = 70%

### Treatment strategies

**Fig. 3:**
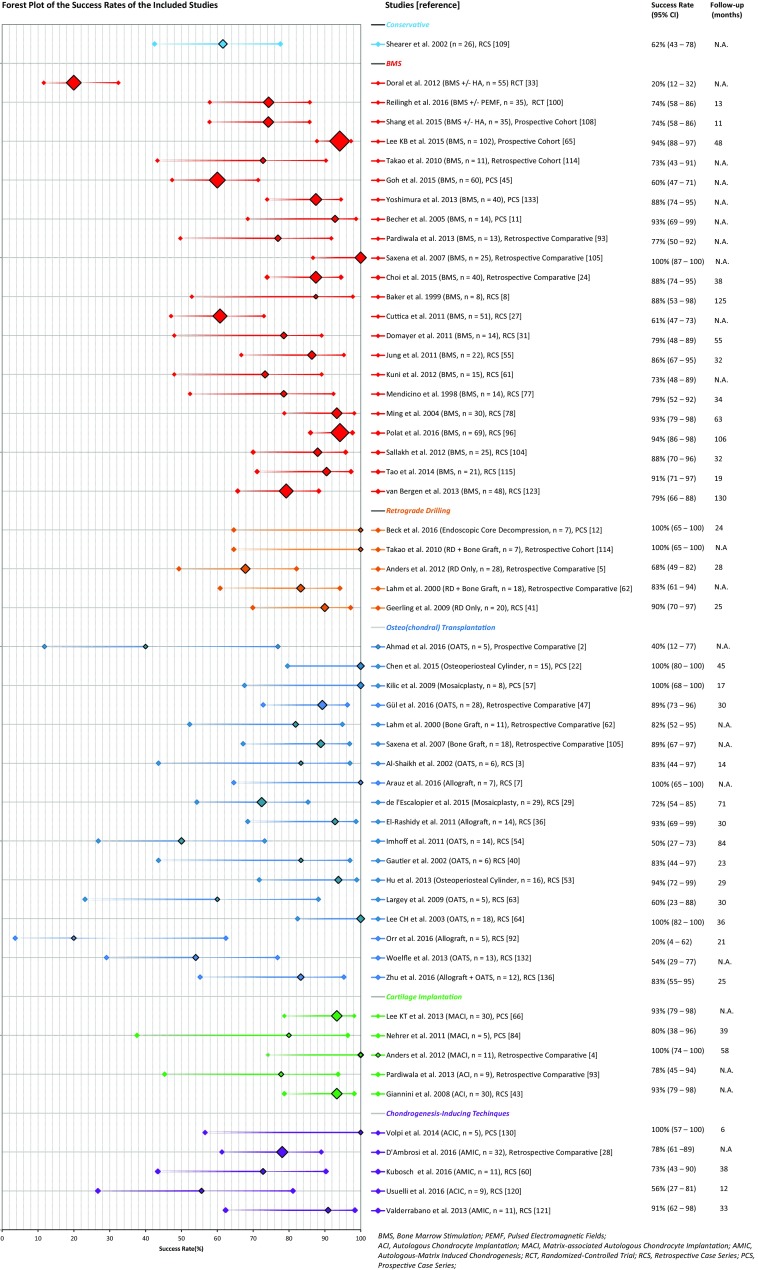
Forest plot of all included studies with the success rates and the corresponding 95% confidence interval per separate study (sorted on treatment strategy group, methodological quality and alphabetical order accompanied by number of ankles and mean follow-up duration; the size of the diamond representing the success rate is adjusted for the number of ankles included in the publications)

**Fig. 4:**
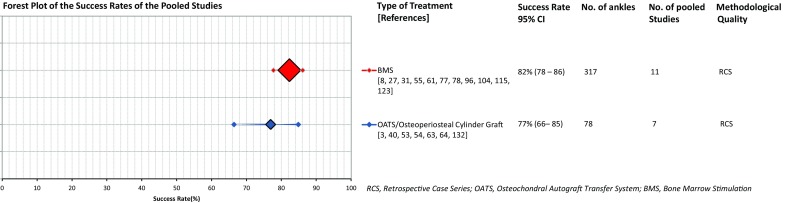
Forest plot of the pooled success rates of different treatment strategies with the corresponding 95% confidence intervals (accompanied by the total number of ankles and total number of studies included in the pooled group, and the corresponding methodological quality; the size of the diamond representing the pooled success rate is adjusted for the number of ankles included)

The different treatment strategies were divided into six corresponding treatment groups. It was deemed methodologically appropriate to perform a simplified pooling method for the largest groups of those publications with corresponding methodological nature (i.e. retrospective case series together) in the groups of BMS and osteo(chondral) transplantation—more specifically OATS and an osteoperiosteal cylinder graft insertion. No studies describing a mosaicplasty procedure were included in this pooling group as mosaicplasty uses multiple graft insertion procedures applied for the treatment of larger talar defects which is in contrast to the classic OATS procedure. Consequently, pooling the mosaicplasty studies was not appropriate. The forest plot describing the clinical results in percentages per separate study in their corresponding treatment group is presented in Fig. 3, and the forest plot describing the results of the simplified pooling method is presented in Fig. 4.

### Non-operative

The objective of non-operative treatment is to unload the damaged cartilage potentially resolving accumulated oedema within the joint.

One retrospective case series study investigated solely chronic-type V cystic lesions as classified by Loomer et al. [68, 109]. Non-operative treatment consisted of continuation of activities “as tolerated” [109]. Mean symptom duration, mean follow-up, patient satisfaction scores and pre-operative OCD size could not be recorded. Eventually, in 16 out of 26 patients conservative treatment yielded successful results, which corresponded to a success rate of 62% [CI 43–78%] (Fig. 3) [109].

### Bone marrow stimulation (debridement and/or drilling)

BMS consists of debriding the OCD after which additional microfracturing or antegrade drilling can be performed establishing openings into the subchondral bone. This disrupts intraosseous vessels introducing blood and bone marrow cells into the OCD allowing a clot of scar tissue to form resulting in fibrocartilaginous tissue. Supplementary, one can administer hyaluronic acid (HA) injections acting as a synovial lubricator targeting pain levels and inflammatory cytokine concentrations [81, 112]. Another possibility is the use of pulsed electromagnetic fields (PEMF) [1, 17, 26, 91, 103, 129].

Twenty-two studies describing the results of BMS for 747 ankles were identified [8, 11, 24, 27, 31, 33, 45, 55, 61, 65, 77, 78, 93, 96, 100, 104, 105, 108, 114, 115, 123, 133]. There were two RCTs, two prospective cohort studies and one retrospective cohort study, three prospective case series, three retrospective comparative studies and eleven retrospective case series. This shows the great heterogeneity in methodological nature of the studies within this group. The means of the symptom duration of these studies ranged from 4 to 49 months, and the range of the means of the follow-up duration in months was as follows: 10–143 months (Fig. 3). For 194 ankles data on Berndt and Harty [15] staging could be extracted: 23, 31, 33 and 13% were affected by stage I, II, III and IV lesions, respectively [8, 24, 65, 78]. The means of the pre-operative size of the talar OCD ranged from 1.0 to 1.7 cm^2^ [24, 65, 96, 105, 108, 115]. The success percentages of the separate studies corresponding to the BMS group ranged from 20 to 100% [CI 12–100%] (Fig. 3) [8, 11, 24, 27, 31, 33, 45, 55, 61, 65, 77, 78, 93, 96, 100, 104, 105, 108, 114, 115, 123, 133]. There were eleven studies within the BMS group that all investigated the patients in a retrospective case series setting, making it methodologically appropriate to perform a simplified pooling method for this subgroup [8, 27, 31, 55, 61, 77, 78, 96, 104, 115, 123]. It contained 317 talar OCDs yielding a pooled success rate of 82% [CI 78–86%] (Fig. 4).

### Retrograde drilling

Retrograde drilling (RD) is a non-transarticular procedure preventing injury to the articular cartilage. Consequently, the technique is primarily used when defects contain a relatively small amount of articular cartilage damage or when it is challenging to reach the OCD via the common arthroscopic portals. The aim is to revascularise the subchondral bone and induce novel bone formation. Additional procedures one can administer are cancellous bone grafts.

Five studies with a total of 80 ankles having undergone retrograde drilling were identified [5, 12, 41, 62, 114]. One prospective case series, one retrospective cohort study, one retrospective case series and two retrospective comparative studies were identified. Therefore, due to the heterogeneity this did not allow for pooling. Furthermore, concerning symptom duration, Berndt and Harty [15] staging and sizes of the talar OCDs, there was insufficient information to provide data on ranges of means reported in the cited literature. The range of the means of follow-up duration was 24–28 months (Fig. 3). The success percentages in this treatment group ranged from 68 to 100% [CI 49–100%] (Fig. 3) [5, 12, 41, 62, 114]. Included in this range were two studies that implemented cancellous bone grafting additional to retrograde drilling with mean success rates ranging from 83 to 100% [CI 61–100%] and two studies that performed retrograde drilling (range 68–90%, CI 49–97%, Fig. 3) [5, 41, 62, 114]. One study by Beck et al. [12] investigated a transtalar endoscopic core decompression combined with the injection of synthetic osteoconductive bone graft substitute. It included 7 patients and yielded a success rate of 100% (Fig. 3) [CI 65–100%].

### Osteo(chondral) transplantation

A number of osteo(chondral) transplantation techniques exist to treat talar OCDs: osteochondral autograft transfer systems (OATS), mosaicplasty, (autogenous) bone grafting, autologous osteoperiosteal cylinder grafting and an osteochondral allograft transfer. The procedures consist of debriding the degenerated cartilage, the fibrous tissue and the necrotic subchondral bone, after which the osteo(chondral) grafts are harvested and subsequently implemented into the remaining OCD. The aim is to achieve a higher-quality restoration of the functional unit of the subchondral bone plate including the articular cartilage.

Eighteen studies were identified, which included a total of 230 primary OCDs [2, 3, 7, 22, 29, 36, 40, 47, 53, 54, 57, 62–64, 92, 105, 132, 136]. There were two prospective case series, one prospective comparative study, three retrospective comparative studies and twelve retrospective case series. This did not allow for subsequent overall osteo(chondral) transplantation group pooling. It was not possible to extract sufficient information on the symptom duration, patient subjective satisfaction scores and staging of the defect. The range of the means of the follow-up duration was 14–84 months, and the range of the means of the sizes per particular study 1.0 to 2.4 cm^2^ [3, 22, 36, 40, 47, 57, 64, 92]. The range of the success percentages per study for the treatment strategy group of osteo(chondral) transplantation was 20 to 100% [CI 4–100%] (Fig. 3) [2, 3, 7, 22, 29, 36, 40, 47, 53, 54, 57, 62–64, 92, 105, 132, 136]. The range of the means of the success percentage per separate publication for the OATS group was 40–100% [CI 12–100%] (Fig. 3), for the mosaicplasty group 72–100% [CI 54–100%] and for one study that combined an OATS and an allograft procedure it was 83% [CI 55–95%] (Fig. 3) [2, 3, 29, 40, 47, 54, 57, 63, 64, 132, 136]. After extracting data on donor-site morbidity of 93 primary and secondarily treated talar OCDs by OATS, it became clear that 32% of the participants showed some form of donor-site knee joint morbidity [2, 3, 29, 40, 63, 64]. Two studies including 31 ankles researched an osteoperiosteal cylinder graft and reported mean success percentages of 94–100% [CI 72–100%] (Fig. 3) [22, 53]. Three studies—with in total 19 ankles—investigated the clinical effectiveness of a fresh allograft transplantation, and the success rates ranged from 20 to 100% [CI 4–100%] [7, 36, 92]. Additionally, there were two studies performing implementation of cancellous bone grafting into 29 debrided talar OCDs [62, 105]. In this group the success rate ranged from 82 to 89% [CI 52–97%] (Fig. 3) [62, 105]. It was possible to perform a simplified pooling method for those studies with a retrospective case series setting investigating an OATS procedure and an osteoperiosteal cylinder graft procedure, and this group of 78 treated talar OCDs yielded a pooled success rate of 77% [CI 66–85%] (Fig. 4) [3, 40, 53, 54, 63, 64, 132].

### Cartilage implantation

Cartilage implantation techniques aim at regenerating tissue with hyaline-like type II cartilage. Generally, in two-step procedures viable chondrocytes are isolated from a donor site, after which the chondrocytes are cultivated and expanded in a laboratory medium. The cultured chondrocytes are then implanted into the excised lesion. When applying the ACI procedure, a periosteal tissue cover is used after expansion of isolated chondrocytes, whereas MACI replaces the periosteal cover by a collagen type 1–3 or Hyalograft C membrane [42]. The latter has the advantage that there is no need for an additional donor site and potentially delivers more viable cells to the OCD [80].

Five studies including 85 ankles investigating cartilage implantation were identified [4, 43, 66, 84, 93]. Two prospective case series, two retrospective comparative studies and one retrospective case series were included in this group. The authors decided not to perform a simplified pooling method. There was insufficient homogeneity and substantial missing data to report mean symptom duration, patient subjective satisfaction scores and staging of the defect. Concerning follow-up duration, it was possible to extract data from two studies, yielding a range of the means of follow-up of 39–58 months (Fig. 3) [4, 84]. From four studies information on talar OCD size could be extracted, which yielded a range of 1.6–1.9 cm^2^ [4, 43, 66, 84]. The success rate ranged from 78 to 100% [CI 45–100%] (Fig. 3) [4, 43, 66, 84, 93]. From these five studies, there were two investigating ACIs [43, 93]. The range of the success rate was 78–93% [CI 45–98%] (Fig. 3) [43, 93]. The other three publications performed a MACI procedure with a total of 46 ankles, and the success percentages ranged from 80 to 100% [CI 38–100%] as illustrated in Fig. 3 [4, 66, 84].

### Chondrogenesis-inducing techniques (CITs)

CITs aim at the repair of a bone-cartilage lesion by means of a combined single-step procedure and can be applied for larger, cystic OCDs [13, 14]. The goal is to induce chondrogenesis, and in case of an adjusted autologous matrix-induced chondrogenesis (AMIC) procedure, spongiosa bone—rich in mesenchymal stem cells—is implanted into the defect [20]. Thereafter, an acellular collagen I/III matrix is glued onto the defect. In case of an autologous collagen-induced chondrogenesis (ACIC) procedure, the debrided defect is filled with a mixture of synthetic fibrin glue and collagen gel-based matrix.

Five publications describing the results of 68 ankles treated by CIT were identified [28, 60, 120, 121, 130]. One study was a prospective case series, one was a retrospective comparative study, and the other three were retrospective case series, which discouraged pooling. There was no sufficient data to allow a presentation of the symptom duration, patient subjective satisfaction scores, staging and sizes of the defect. The range of the means of follow-up duration was 6–38 months (Fig. 3). The range of the success rate was 56–100% [CI 27–100%] (Fig. 3) [28, 60, 120, 121, 130]. For the AMIC procedures, the range of success percentages was 73 to 91% [CI 43–98%] (Fig. 3) [28, 60, 121]. Volpi et al. [130] and Usuelli et al. [120] described the results of ACIC, and the means of the success rate ranged from 56 to 100% [CI 27–100%] (Fig. 3).

## Discussion

To the best of our knowledge, this is the first systematic review investigating the effectiveness of all treatment options for solely *primary* talar OCDs in adults. The most important finding of the present study is that although aiming at the application of the most appropriate and complete methodology, none of the interventions showed any definite clinical superiority over the others. This was caused by the observed heterogeneity in methodological nature of the studies and the variety in success rates, both intra-treatment strategy group-wise and inter-treatment strategy group-wise. Additionally, performing a simplified pooling method for retrospective case series studies in the BMS group and in the osteo(chondral) transplantation group yielded comparable pooled success rates.

The main finding is partially in contrast to the one derived from the research by Zengerink et al. [135] which concluded that BMS is the most effective treatment strategy for talar OCDs. This systematic review from 2010, however, included both primary and non-primary talar OCDs, which potentially affected the results and the conclusions based on them. It should be acknowledged that the most important finding of the present study was not a consequence of the methodology, as it aspired to include as many suitable articles as possible by not excluding particular treatment strategies—in contrast to previous reviews [32, 85]—and by adhering to a strict author contact protocol.

BMS was the most studied intervention for primary talar OCDs indicating that it is the most frequently practised treatment option for primary talar OCDs worldwide. This is due to the fact that BMS is a relatively inexpensive intervention compared to implantation techniques, has low morbidity, a quick recovery and a fast return to sports. This was shown by studies conducted by Saxena et al. [105] and Reilingh et al. [100] presenting return to sports times ranging from 15 to 17 weeks. The two most recent systematic reviews on BMS reported success rates of 80 and 86% [32, 135]. When pooling eleven BMS studies, a pooled success rate of 82% was calculated [CI 78–86%] (Fig. 4). As this success rate is comparable to the success rate of the pooled retrospective case series design studies in the osteo(chondral) transplantation group describing the results of OATS and an osteoperiosteal cylinder graft insertion (77% [CI 66–85%]), it is difficult to assess which surgical treatment strategy is clinically superior, thereby supporting the most important finding of the present study. Important factors play a vital role in the success of the clinical outcome after BMS. BMS does not aim at preserving a hyaline cartilage layer but rather promotes the formation of a fibrin clot subsequently becoming fibrocartilage or cartilage/collagen type I, which may then decrease in quality over time, resulting in osteoarthritic changes [70, 88, 89]. Moreover, research indicates that deterioration of the natural congruency of the ankle joint occurs as cartilage type I demonstrates inferior wear characteristics in comparison with hyaline cartilage (cartilage/collagen type II) being associated with the degradation of a repaired articular surface [74, 98, 111]. However, long-term studies have not yet confirmed this [37, 123]. A clear correlation between inferior clinical outcomes and follow-up duration concerning the included studies in this review was not observed either, possibly due to the fact that it was not possible to gather data on mean follow-up durations from all included studies. Concerning pre-operative size and clinical outcome after BMS, a study from Choi et al. [25] including 120 primary ankles indicated that there is a definite cut-off point, that is, 1.5 cm^2^, as a prognostic influence on the risk of clinical failure. A more recent study by Ramponi et al. [99] shows that the cut-off point might be lower, around the size of 107 mm^2^. In our review, the range of the means of the reported pre-operative size for the BMS studies was 1.0 to 1.7 cm^2^ suggesting that BMS is indeed administered for smaller primary defects. The reported success rates of BMS therefore suggest that BMS could be regarded as a fair treatment strategy for the smaller primary defects.

As an alternative to BMS, a number of treatment options have focused on preserving hyaline cartilage and treating larger defects. The consensus that most of these interventions are considered as suitable treatment options when primary surgery to the OCDs has failed explains why there was a relatively lower number of patients included in these particular treatment groups. Furthermore, a number of publications on the osteochondral autograft system had to be excluded. Studies by Hangody et al. [50] and Fraser et al. [38] have yielded promising results, but were excluded as legal cases needed to be reopened for data provision.

Interestingly, only one study described the results of non-operative treatment implying that since 1996 studies have focused on developing novel *surgical* treatment options [109]. Likely, this is due to the poor success rates of non-operative treatments reported before 1996 [16, 102]. Although only twenty-six conservatively treated ankles were included in our review—with a success percentage of 62% [CI 43–78%]—it is still recommended that initial treatment of symptomatic OCDs should consistently commence with a conservative protocol.

The AOFAS score was the most frequently used clinical score among the included studies. Sierevelt et al. [110] indicated that there are some concerns regarding this outcome score. A significant part of the 100 points depends on patient subjective outcomes introducing bias to the interpretation of the calculated success rates, as a high-level athlete would subjectively rate his or her surgery more critically than the average patient included in our systematic review. Moreover, the AOFAS score is not officially validated for the clinical evaluation of the treatment of talar OCDs. Therefore, future research should focus on developing a for-talar-OCD-validated outcome scale, in order to increase the homogeneity and uniformity in outcome assessment.

As the review shows that in 71% of the cases a history of ankle trauma was reported, it is as important to focus on prevention strategies as focusing on effective surgical treatment measures. Progression has been made regarding the development of cost-effective prevention programs for lateral and medial ankle sprains, for example by Verhagen [127] through the development of a mobile application system.

Furthermore, the analysis concerning methodological quality showed that a high number of studies included were of low methodological quality, except for two included RCTs [33, 100]. This underlines that the necessity for more sufficiently powered randomised studies is of paramount importance. Future research should therefore focus on conducting more randomised comparative clinical trials with uniform methodology and extended follow-up times. BMS should be compared to newly developed promising treatment options that focus on preserving hyaline cartilage and preventing the development of additional clinical complaints, such as donor-site morbidities observed in patients undergoing an OATS procedure. A possible future direction for such a promising treatment strategy is the internal fixation surgeries. In small patient series, these have been shown to induce a significant clinical improvement, possibly because these aim at preserving hyaline cartilage [56, 58].

There were a number of limitations concerning the present review. Firstly, the low quality of the included studies and the substantial heterogeneity regarding methodology account as major limitations. Additionally, separate success rates were calculated based on different scoring systems, as the AOFAS score was not always available for statistical analysis. Due to this, it was not possible to perform the conventional measure of summarising estimates of effectiveness. Concerning patient characteristics there was heterogeneity observed in the patient population. It was not possible to collect data concerning mean follow-up duration on all studies included, as these were not provided in all cases. Another limitation of the study is that it was not possible to perform a formal meta-analysis utilising mixed-effects logistics regression in order to compare between treatment groups. Regarding the BMS group and the studies within the osteo(chondral) transplantation group, those publications that had utilised a retrospective case series setting were pooled. This implies that the evidence retrieved from this simplified pooling method is based on lower level of evidence and may therefore contain methodological bias indicating that the pooled calculated success rates should not be used for decision of a particular treatment technique for talar OCDs, but merely be applied to inform patients in the process of explaining the expected success percentages of a particular treatment strategy. Moreover, the pooled success rate of the osteo(chondral) transplantation group combined studies reporting the effects of OATS procedures and an osteoperiosteal cylinder procedure possibly introducing some form of heterogeneity in this group as the type of grafts inserted in the OATS group was slightly different from the ones in the osteoperiosteal cylinder group [3, 40, 53, 54, 63, 64, 132]. The strengths of the present review are the inclusion of solely primary lesions, the thorough reference selection and the quality assessment of the included studies. Another major strength is the extensive corresponding author contact protocol regarding additional data retrieval and further clarification on methodology of included studies.

The clinical relevance of the present systematic review is that the separate and pooled success rates for the different surgical and non-surgical management options can be utilised to inform patients about the expected success percentages when undergoing treatment for primary talar osteochondral defects, which will facilitate the shared decision-making process between patients and physicians.

## Conclusions

In conclusion, the present systematic review shows that none of the interventions for the treatment of primary osteochondral defects to the talus showed clinical superiority over another or others. A simplified pooling method for eleven retrospective case series in the BMS group yielded a success rate of 82% [CI 78–86%], and for the seven combined OATS and osteoperiosteal cylinder graft studies the pooled success rate was calculated to be 77% [CI 66–85%]. A high number of studies with low methodological quality were included, and heterogeneity in methodological nature of the studies and variety in reported success rates was observed. As a consequence, future research should focus on conducting sufficiently powered prospective investigations in a randomised comparative clinical trial setting using outcome scores validated for the treatment of talar OCDs.

## Appendix 1

Full electronic search strategy used in this systematic review

**1. PubMed**

**Table:**

#	**Searches**	**Results**
1	“Osteochondritis Dissecans”[Mesh]	Total number of results 1996–2017: 1053 hits
2	Osteochondritis dissecans[tiab] OR osteochondrosis dissecans[tiab] OR osteochondrolysis[tiab] OR OCD[tiab] OR OLT[tiab]	
3	(osteochondral[tiab] OR chondral[tiab] OR transchondral[tiab] OR cartilage*[tiab]) AND (defect*[tiab] OR lesion*[tiab])	
4	#1 OR #2 OR #3	
5	“Talus”[Mesh]	
6	talus[tiab] OR talar*[tiab] OR ankle[tiab]	
7	#5 OR #6	
8	#4 AND #7	

**2. EMBASE (OVID)**

**Table:**

#	**Searches**	**Results**
1	(osteochondritis dissecans/or (osteochondritis dissecans or osteochondrosis dissecans or osteochondrolysis or OCD or OLT).ti,ab,kw. or ((osteochondral or chondral or osteochondral or transchondral or cartilage*) adj3 (defect* or lesion*)).ti,ab,kw.) and (talus/or (talus or talar* or ankle).ti,ab,kw.)	1475
2	limit 1 to yr = “1996–2017”	1220

**3. COCHRANE LIBRARY**

**Table:**

#	**Searches**	**Results**
1	MeSH descriptor: [Osteochondritis Dissecans] explode all trees	8
2	osteochondritis dissecans or osteochondrosis dissecans or osteochondrolysis or OCD or OLT:ti,ab,kw (Word variations have been searched)	1188
3	(osteochondral or chondral or transchondral or cartilage*) and (defect* or lesion*):ti,ab,kw (Word variations have been searched)	343
4	#1 or #2 or #3	1516
5	MeSH descriptor: [Talus] explode all trees	33
6	talus or talar* or ankle:ti,ab,kw (Word variations have been searched)	5266
7	#5 or #6	5266
8	#4 and #7, Publication Year from 1996 to 2017, in Cochrane Reviews (Reviews and Protocols), Other Reviews and Trials	33

## Appendix 2

Grading scale as proposed by Thompson and Loomer [118]

**Table:**

Rating	Pain	Function	Examination	X-ray
Good	None	No restriction on activities	Normal	Normal
Fair	Occasionally with activity	Some limitation of activities	Mild swelling; slight decrease in motion	Minimal change
Poor	As before or worse	Moderate restriction of activities	Arthrosis, i.e. increased swelling and crepitus	Degenerative change
